# Analysis of Cardiovascular Complications During Delivery Admissions Among Patients With Systemic Lupus Erythematosus, 2004-2019

**DOI:** 10.1001/jamanetworkopen.2022.43388

**Published:** 2022-11-29

**Authors:** Salman Zahid, Mohamed S. Mohamed, Heba Wassif, Noreen T. Nazir, Sadiya S. Khan, Erin D. Michos

**Affiliations:** 1 Department of Medicine, Sands-Constellation Heart Institute, Rochester General Hospital, Rochester, New York; 2 Division of Cardiology, Cleveland Clinic, Cleveland, Ohio; 3 Division of Cardiology, University of Illinois at Chicago, Chicago, Illinois; 4 Division of Cardiology, Northwestern University Feinberg School of Medicine, Chicago, Illinois; 5 Division of Cardiology, Johns Hopkins University School of Medicine, Baltimore, Maryland

## Abstract

**Importance:**

Individuals with systemic lupus erythematosus (SLE) have an increased risk of pregnancy-related complications. However, data on acute cardiovascular complications during delivery admissions remain limited.

**Objective:**

To investigate whether SLE is associated with an increased risk of acute peripartum cardiovascular complications during delivery hospitalization among individuals giving birth.

**Design, Setting, and Participants:**

This population-based cross-sectional study was conducted with data from the National Inpatient Sample (2004-2019) by using *International Classification of Diseases, Ninth Revision, Clinical Modification *(*ICD-9-CM*) or *International Statistical Classification of Diseases and Related Health Problems, Tenth Revision, Clinical Modification *(*ICD-10-CM*) codes to identify delivery hospitalizations among birthing individuals with a diagnosis of SLE. A multivariable logistic regression model was developed to report an adjusted odds ratio (OR) for the association between SLE and acute peripartum cardiovascular complications. Data were analyzed from May 1 through September 1, 2022.

**Exposure:**

Diagnosed SLE.

**Main Outcomes and Measures:**

Primary study end points were preeclampsia, peripartum cardiomyopathy, and heart failure. Secondary end points included ischemic and hemorrhagic stroke, pulmonary edema, cardiac arrhythmias, acute kidney injury (AKI), venous thromboembolism (VTE), length of stay, and cost of hospitalization.

**Results:**

A total of 63 115 002 weighted delivery hospitalizations (median [IQR] age, 28 [24-32] years; all were female patients) were identified, of which 77 560 hospitalizations (0.1%) were among individuals with SLE and 63 037 442 hospitalizations (99.9%) were among those without SLE. After adjustment for age, race and ethnicity, comorbidities, insurance, and income level, SLE remained an independent risk factor associated with peripartum cardiovascular complications, including preeclampsia (adjusted OR [aOR], 2.12; 95% CI, 2.07-2.17), peripartum cardiomyopathy (aOR, 4.42; 95% CI, 3.79-5.13), heart failure (aOR, 4.06; 95% CI, 3.61-4.57), cardiac arrhythmias (aOR, 2.06; 95% CI, 1.94-2.21), AKI (aOR, 7.66; 95% CI, 7.06-8.32), stroke (aOR, 4.83; 95% CI, 4.18-5.57), and VTE (aOR, 6.90; 95% CI, 6.11-7.80). For resource use, median (IQR) length of stay (3 [2-4] days vs 2 [2-3] days; *P* < .001) and cost of hospitalization ($4953 [$3305-$7517] vs $3722 [$2606-$5400]; *P* < .001) were higher for deliveries among individuals with SLE.

**Conclusions and Relevance:**

This study found that SLE was associated with increased risk of complications, including preeclampsia, peripartum cardiomyopathy, heart failure, arrhythmias, AKI, stroke, and VTE during delivery hospitalization and an increased length and cost of hospitalization.

## Key Points

**Question:**

Is systemic lupus erythematosus (SLE) among pregnant individuals associated with an increased risk of acute cardiovascular complications during delivery hospitalizations?

**Findings:**

In this cross-sectional study of 63 million delivery admissions, including 77 560 admissions among individuals with SLE, the disease was associated with an increased risk of developing peripartum cardiovascular complications, including preeclampsia, peripartum cardiomyopathy, heart failure, and cardiac arrhythmias, during delivery hospitalization.

**Meaning:**

This study found that SLE was an independent risk factor associated with many peripartum cardiovascular complications at the time of delivery admission.

## Teaser

This cohort study investigates the association of systemic lupus erythematosus with cardiovascular complications during delivery admissions from 2004 to 2019.

## Introduction

Systemic lupus erythematosus (SLE) is a chronic autoimmune disease with myriad clinical manifestations involving the musculoskeletal system, cardiovascular (CV) system, central nervous system (CNS), and kidneys.^1^ Previously, kidney and CNS complications were the leading factors associated with mortality in SLE.^2,3^ However, with the improvement in immunomodulatory therapy, survival rates have increased, along with an increase in CV morbidity and mortality rates.^4,5^ It is well established that individuals with SLE have approximately 2-fold to 6-fold higher risk of major CV events compared with the general population, and CV events account for 30% of mortality 5 years after diagnosis.^6,7,8,9^ Individuals with SLE have a higher prevalence of factors traditionally associated with CV risk, such as obesity, hypertension, and diabetes, which only partially contribute to this population’s increased risk of adverse CV events.^10,11,12,13^ Inflammation is thought to be a central contributor to increased CV risk.^14^ Polycystic ovary syndrome (PCOS) is also associated with SLE^15^ and with CV risk.^16^

Pregnant individuals with SLE are at increased risk for adverse maternal and fetal outcomes.^17,18^ Although the associations of SLE with long-term CV risks are well-described, data on the associations of SLE with acute CV complications at the time of delivery are less well established. Given increasing maternal mortality rates in the United States owing to peripartum CV complications,^19^ it is imperative to identify novel risk factors to implement preventive strategies aimed at reducing mortality rates. Hence, we aimed to study the association of SLE with trends, outcomes, and risk factors of CV complications during delivery hospitalizations using the US nationwide National Inpatient Sample (NIS) database.

## Methods

Rochester General Hospital determined that this cross-sectional study was exempt from institutional review board approval and informed consent because NIS data are deidentified and publicly available. We used the Strengthening the Reporting of Observational Studies in Epidemiology (STROBE) reporting guideline to report study findings. The specific data supporting this study’s findings are available from the corresponding author upon request.

### Study Data

This study used data from the NIS database from 2004 to 2019. The NIS is managed by the Agency for Healthcare Research and Quality through a federal-state-industry partnership called the Healthcare Cost and Utilization Project (HCUP).^20^ The NIS contains administrative claims data from more than 7 million inpatient hospitalizations annually in 47 participating states and the District of Columbia, representing more than 97% of the US population. Sample weights are provided by NIS to calculate national estimates. Because NIS data are compiled annually, they can be used for analysis of disease trends over time using trend weights compiled by the HCUP. For the cost of care, charge to cost ratios supplied by HCUP and derived from the Centers for Medicare & Medicaid Services were applied to total hospital charges. Data on race and ethnicity were collected by HUCP participating organizations.^21^ Race and ethnicity categories in the database were Asian or Pacific Islander, Black, Hispanic, Native American, White, and race and ethnicity not listed or multiracial (combined as other in this study because multiracial was defined as other in the database). Race and ethnicity were evaluated in the study given that there is significant racial disparity with respect to maternal health outcomes at the time of delivery admissions.

### Study Design and Data Selection

We analyzed NIS data using *International Classification of Diseases, Ninth Revision, Clinical Modification* (*ICD-9-CM*) and *International Statistical Classification of Diseases, Tenth Revision, Clinical Modification* (*ICD-10-CM*) claims codes. We first identified delivery hospitalizations for adult patients (ages ≥18 years) using *ICD-9-CM* and *ICD-10-CM* codes (eTable 1 in the Supplement). Among selected patients, we used *ICD-9-CM* code 7100 and *ICD-10-CM* code M32 to identify delivery hospitalizations with SLE. All diagnosis fields were queried to select and categorize the study population. A study overview and detailed methods flow chart are presented in eFigure 1 and eFigure 2 in the Supplement, respectively.

### Study End Point

The primary study end points were preeclampsia, peripartum cardiomyopathy (PPCM), and heart failure. Secondary end points included ischemic and hemorrhagic stroke, pulmonary edema, cardiac arrhythmias, acute kidney injury (AKI), acute venous thromboembolism (VTE), length of stay, and cost of hospitalization. Associated procedures and complications were identified using *ICD-9-CM* and *ICD-10-CM* codes. Due to the low number of hospitalizations with eclampsia in the sample, these outcomes were categorized as preeclampsia (eTable 1 in the Supplement).

### Statistical Analysis

Descriptive statistics were presented as frequencies with percentages for categorical variables and as medians with IQRs for continuous variables. Baseline characteristics were compared using a Pearson χ^2^ test or Fisher exact test as appropriate for categorical variables and the Mann-Whitney U test for continuous variables.

Unadjusted odds ratios (ORs) were derived using the Cochran-Mantel-Haenszel test. For the continuous length of stay and cost of hospitalization variables, linear regression was performed to compute effect sizes. A multivariable logistic regression model was fitted to test the association of SLE with in-hospital outcomes, adjusted for age, race and ethnicity, hospital region, prepregnancy comorbidity (ie, chronic hypertension, dyslipidemia, heart failure, chronic kidney disease, coronary artery disease, PCOS, and obesity), smoking, multiple gestation, gestational diabetes (GD), cesarean delivery, median household income, and primary insurance (eMethods 1 in the Supplement). We performed a supplementary analysis and retested associations using the previously mentioned multivariable logistic regression model with additional adjustment for preeclampsia and eclampsia and calendar year. This was done to investigate whether SLE was independently associated with acute CV complications after accounting for these factors. Multicollinearity diagnostic testing was performed for this model. A tolerance of 0.2 or less and a variance inflation factor (VIF) of and 5 or more were taken as suggestive of the potential existence of multicollinearity. Given the known association between SLE and preeclampsia and eclampsia,^17^ we also performed a sensitivity analysis by excluding hospitalizations with preeclampsia or eclampsia; we retested the evaluation using the previously mentioned multivariable logistic regression model to investigate whether SLE was associated with acute CV complications in the absence of preeclampsia and eclampsia. Similarly, we performed a supplementary analysis after excluding hospitalizations with PCOS, GD, preexisting coronary artery disease, chronic heart failure, and chronic kidney disease to retest the association between SLE and peripartum CV complications.

We evaluated socioeconomic disparities and assessed potential independent variables associated with our primary outcome of preeclampsia among individuals with SLE adjusted for age, race and ethnicity, chronic hypertension, dyslipidemia, obesity, GD, PCOS, median household income, and primary insurance. For trend analysis, binary logistic regression adjusted for age was used to calculate a trend *P* value (eMethods 2 in the Supplement).

Covariates were selected based on a prior literature review. Missing values present in the data set are reported in Table 1. These were predominantly present in the race and ethnicity variable (8 583 640 of 63 115 002 hospitalizations [13.6%]) and were recoded as the other category. Given the overall a low number of missing data (<1.6%) in other variables (eg, 98 037 of 63 115 002 hospitalizations [0.2%] for the primary insurance variable ), we used listwise deletion and did not include missing data in the logistic regression analysis.

**Table 1.** Characteristics of Delivery Hospitalizations With and Without SLE

Characteristic	Hospitalizations, No. (%) (N = 63 115 002)		*P* value
	Without SLE (n = 63 037 442)	With SLE (n = 77 560)	
Demographics			
Age, median (IQR), y	28 (24-32)	30 (26-34)	<.001
Race and ethnicity			
Asian or Pacific Islander	3 011 659 (5.5)	3632 (5.2)	<.001
Black	7 726 808 (14.2)	16 397 (23.7)	
Hispanic	11 983 724 (22.0)	12 975 (18.7)	
Native American	4,21 104 (0.8)	559 (0.8)	
White	28 817 995 (52.8)	32 909 (47.5)	
Other^a^	11 076 152 (17.6)	11 088 (14.3)	
Hospital region			
Northeast	10 448 057 (16.6)	14 649 (18.9)	<.001
Midwest	13 404 386 (21.3)	14 669 (18.9)	
South	23 912 291 (37.9)	30 210 (39.0)	
West	15 272 707 (24.2)	18 032 (23.2)	
Preexisting comorbidity			
PCOS	189 907 (0.3)	461 (0.6)	<.001
GD	2 431 317 (3.9)	3984 (5.1)	<.001
Dyslipidemia	92 010 (0.1)	372 (0.5)	<.001
Chronic hypertension	410 037 (0.7)	3826 (4.9)	<.001
Heart failure	38 908 (0.1)	600 (0.8)	<.001
Chronic kidney disease	8761 (0.0)	870 (1.1)	<.001
Coronary artery disease	7249 (0.0)	184 (0.2)	<.001
Obesity	2 513 158 (4.0)	4565 (5.9)	<.001
Smoking	1 205 023 (1.9)	1784 (2.3)	<.001
Obstetric characteristic			
Multiple gestation	1 212 676 (1.9)	2077 (2.7)	<.001
Cesarean delivery	19 876 453 (31.5)	32 253 (41.6)	<.001
Birth			
Preterm	4 640 784 (7.4)	11 718 (15.1)	<.001
Still	428 020 (0.7)	1291 (1.7)	<.001
Socioeconomic characteristic			
Median household income, percentile			
0-25	16 949 244 (27.3)	20 735 (27.1)	<.001
26-50	15 545 298 (25.1)	17 663 (23.1)	
51-75	15 287 506 (24.7)	18 676 (24.4)	
76-100	14 224 295 (22.9)	19 332 (25.3)	
Missing	1 031 099 (1.6)	1154 (1.5)	
Primary insurance			
Medicare	422 881 (0.7)	3799 (4.9)	<.001
Medicaid	26432835 (42.0)	28 445 (36.7)	
Private	32 367 828 (51.4)	41 543 (53.6)	
Self-pay	1 900 056 (3.0)	1162 (1.5)	
No charge	98 562 (0.2)	64 (0.1)	
Other	1 717 365 (2.7)	2425 (3.1)	
Missing	97 915 (0.2)	122 (0.2)	

Abbreviations: GD, gestational diabetes; PCOS, polycystic ovary syndrome; SLE, systemic lupus erythematosus.

^a^ Other denotes races and ethnicities not listed in database categories or multiracial. Other also includes hospitalizations with missing race and ethnicity data.

All statistical analyses were performed using SPSS statistical software version 27 (IBM). Given the complex survey design of NIS, we applied sample weights, clusters, and strata to generate US national estimates. A 2-sided *P* value < .05 was considered statistically significant. Data were analyzed from May 1 through September 1, 2022.

## Results

### Hospitalization Characteristics of the Study Population

A total of 63 115 002 weighted hospitalizations for deliveries (median [IQR] age, 28 [24-32] years; all were female patients) were identified in the US from 2004 to 2019. Of these 77 560 hospitalizations (0.1%) had a diagnosis of SLE and 63 037 442 hospitalizations (99.9%) did not have a diagnosis of SLE. Detailed baseline characteristics are given in Table 1. Patients with SLE had a higher median (IQR) age of 30 (26-34) years compared with 28 (24-32) years for patients without SLE (*P* < .001). Individuals with vs without SLE were less likely to be White and more likely to be Black. Among individuals with SLE there were 32 909 White individuals (47.5%), 16 397 Black individuals (23.7%), 12 975 Hispanic individuals (18.7%), 3632 Asian or Pacific Islander individuals (5.2%), and 11 088 individuals with other race or ethnicity (14.3%), while among those without SLE, there were 28 817 995 White individuals (52.8%), 7 726 808 Black individuals (14.2%), 11 983 724 Hispanic individuals (22.0%), 3 011 659 Asian or Pacific Islander individuals (5.5%), and 11 076 152 individuals with other race or ethnicity (17.6%). Obesity (4565 hospitalizations [5.9%] vs 2 513 158 hospitalizations [4.0%]; *P* < .001), PCOS (461 hospitalizations [0.6%] vs 189 907 hospitalizations [0.3%]; *P* < .001), GD (3984 hospitalizations [5.1%] vs 2 431 317 hospitalizations [3.9%]; *P* < .001), and dyslipidemia (372 hospitalizations [0.5%] vs 92 010 hospitalizations [0.1%]; *P* < .001) were more frequent in the SLE group compared with the group without SLE. Effect sizes for the association between hospitalization characteristics in patients with SLE vs without SLE are shown in eTable 2 in the Supplement.

### Trends for Prevalence of SLE, CV Complications, PCOS, GD, and Obesity

**Figure 1.**
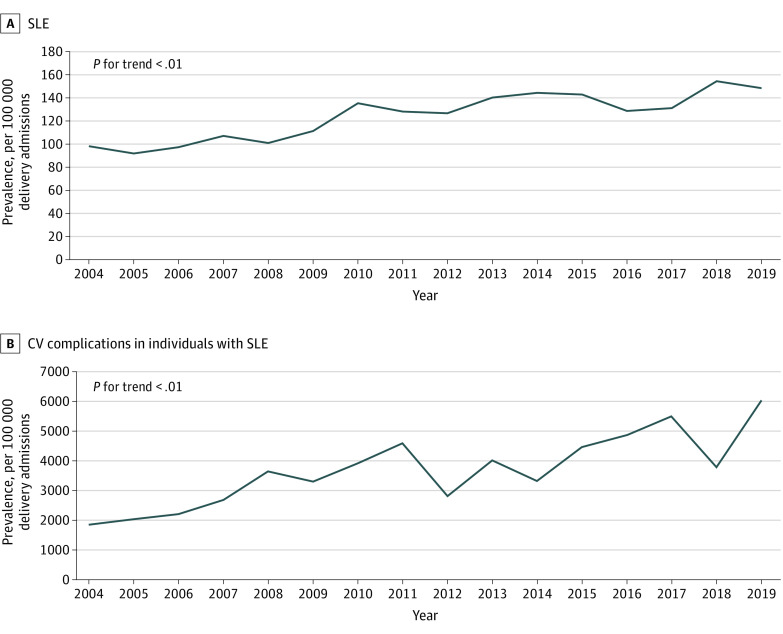
Prevalence of SLE and Acute Cardiovascular Complications CV indicates cardiovascular; SLE, systemic lupus erythematosus.

During the study period, from 2004 to 2019, the prevalence of SLE increased from 98 hospitalizations to 148 hospitalizations per 100 000 deliveries. During the same period, increases in overall acute peripartum CV complication rates were also observed (Figure 1). Moreover, during this period, there was an increase in the prevalence of PCOS from 120 hospitalizations to 1416 hospitalizations per 100 000 hospitalizations with SLE. The prevalence of obesity increased from 29 of 4170 hospitalizations [0.7%] in 2004 to 720 of 5295 hospitalizations [13.6%] in 2019, and GD prevalence increased from 206 of 4170 hospitalizations [4.9%] in 2004 to 415 of 5295 hospitalizations [7.8%] in 2019 (eFigure 3 in the Supplement).

### CV Complications Associated With SLE

Patients with SLE had a higher incidence of CV complications compared with patients without SLE during delivery hospitalizations (Table 2). Patients with SLE had higher rates of preeclampsia (12 605 patients vs 4576 patients per 100 000 hospitalizations; *P* < .001). Similarly, SLE was associated with higher rates of PPCM (304 patients vs 33 patients per 100 000 hospitalizations; *P* < .001). Other CV complications, including stroke, cardiac arrhythmias, and pulmonary edema, were also more common with deliveries in individuals with SLE.

**Table 2.** Complication Rates and Hospital Resource Use in Patients With and Without SLE

Variable	Hospitalizations, No./100 000 hospitalizations (N = 63 115 002)		*P* value
	Without SLE (n = 63 037 442)	With SLE (n = 77 560)	
Complication			
Preeclampsia	4576	12 605	<.001
Peripartum cardiomyopathy	33	304	<.001
Heart failure	44	569	<.001
Acute kidney injury	52	1229	<.001
Stroke	32	267	<.001
Pulmonary edema	38	153	<.001
Cardiac arrhythmias	523	1333	<.001
Venous thromboembolism	37	361	<.001
Hospital resource use, median, (IQR)			
Length of stay, d	2 (2-3)	3 (2-4)	<.001
Cost of hospitalization, $	3722 (2606-5400)	4953 (3305-7517)	<.001

Abbreviation: SLE, systemic lupus erythematosus.

### Odds of In-Hospital Complications

**Figure 2.**
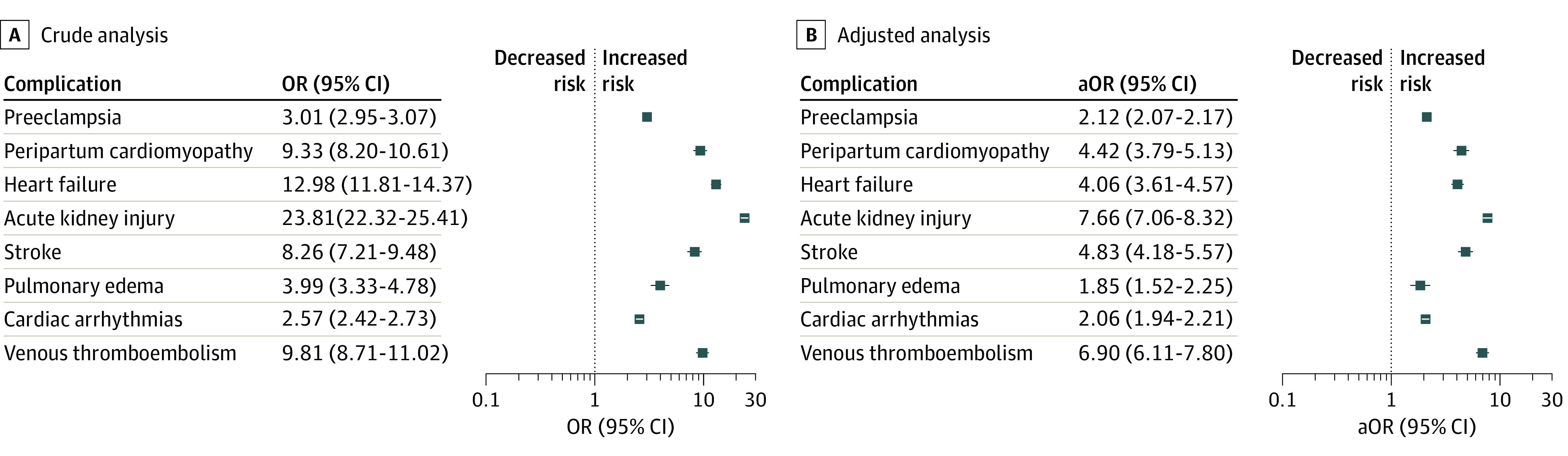
Association of Systemic Lupus Erythematosus With Odds of In-Hospital Complications aOR indicates adjusted odds ratio; OR, odds ratio.

After adjustment for age, race and ethnicity, comorbidities, insurance type, and income level, SLE remained an independent risk factor associated with many CV complications (Figure 2). SLE was associated with higher odds of preeclampsia (adjusted OR [aOR], 2.12; 95% CI, 2.07-2.17; *P* < .001). Similarly, SLE was associated with higher odds of PPCM (aOR, 4.42; 95% CI, 3.79-5.13; *P* < .001), stroke (aOR, 4.83; 95% CI, 4.18-5.57; *P* < .001), pulmonary edema (aOR, 1.85; 95% CI, 1.52-2.25; *P* < .001), AKI (aOR, 7.66; 95% CI, 7.06-8.32; *P* < .001), acute heart failure (aOR, 4.06; 95% CI, 3.61-4.57; *P* < .001), cardiac arrhythmias (aOR, 2.06; 95% CI, 1.94-2.21; *P* < .001), and VTE (aOR, 6.90; 95% CI, 6.11-7.80; *P* < .001). In a supplementary analysis after additional adjustment for preeclampsia, results were similar except for acute heart failure (aOR, 1.20; 95% CI, 0.99-1.45; *P* = .07), which did not reach statistical significance (eTable 3 in the Supplement). No differences in association with study outcomes were observed after adjustment for calendar year (eTable 4 in the Supplement). For individuals with advanced maternal age (>35 years), SLE was associated with acute heart failure (aOR, 1.58; 95% CI, 1.02-2.46; *P* = .04); however, the same association was not seen for maternal age 35 years or younger (eTables 5 and 6 in the Supplement). The sensitivity analysis after exclusion of preeclampsia and eclampsia, PCOS, GD, chronic kidney disease, coronary artery disease, and chronic heart failure mirrored our primary analysis, with no differences in the association of SLE with study outcomes (eTables 7-10 in the Supplement).

### Socioeconomic Disparities and Association of Hospitalization Characteristics With Preeclampsia

**Figure 3.**
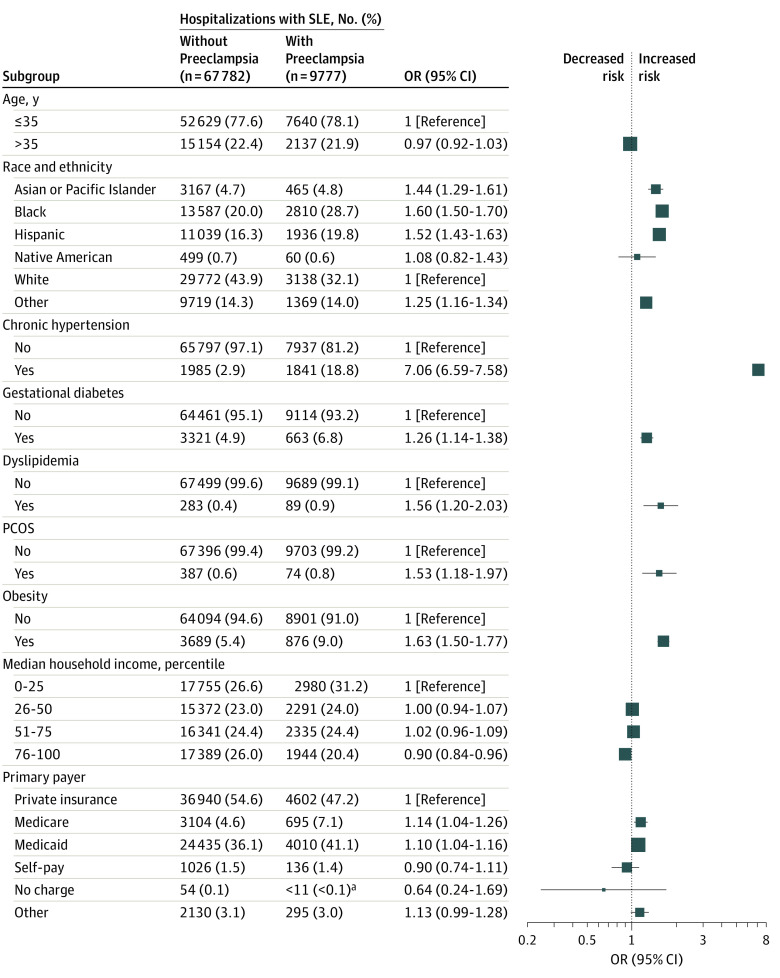
Association of Hospitalization Characteristics With Preeclampsia Among Patients With SLE PCOS indicates polycystic ovary syndrome; SLE, systemic lupus erythematosus. ^a^Observations with counts less than 11 are not reported, per Healthcare Cost and Utilization Project guidelines.

Among individuals with SLE, the factors of chronic hypertension, dyslipidemia, PCOS, GD, obesity, and Black, Hispanic, and Asian or Pacific Islander race or ethnicity were identified as independent risk factors associated with preeclampsia (Figure 3). Individuals with Medicare or Medicaid had the highest odds for preeclampsia compared with private insurance. Individuals in the highest quartile of income had the lowest odds of preeclampsia compared with individuals in the lowest quartile of income. All variables included in the model had a tolerance of more than 0.2 and VIF of less than 5 (eg, hypertension: tolerance, 0.96; VIF, 1.1), indicating the absence of multicollinearity. Detailed hospitalization characteristics and their association with preeclampsia among individuals with SLE are illustrated in Figure 3.

### Resource Use

Median (IQR) length of hospital stay was higher for deliveries among individuals with SLE vs individuals without SLE (3 [2-4] days vs 2 [2-3] days; *P* < .001). Similarly, deliveries for individuals with SLE had a higher median (IQR) cost of hospitalization ($4953 [$3305-$7517] vs $3722 [$2606-$5400]; *P* < .001) (Table 2). Effect sizes for the association between hospitalization resource use in patients with SLE compared with those without SLE are shown in eTable 2 in the Supplement.

## Discussion

This large, contemporary, population-based cross-sectional study, including 63 million delivery hospitalizations in the US, yielded 4 principal findings. First, a diagnosis of SLE was independently associated with higher rates of CV complications during delivery hospitalizations, preeclampsia and eclampsia, PPCM, heart failure, stroke, pulmonary edema, cardiac arrhythmias, AKI, and VTE. Second, SLE during delivery admissions was associated with increased cost and length of delivery hospitalization. Third, the prevalence of obesity, PCOS, and GD in individuals with SLE during delivery hospitalizations increased in the US over a period of 15 years. Fourth, socioeconomic factors of belonging to a minority race or ethnicity group (including Black, Hispanic, and Asian or Pacific Islander groups) and comorbidities, such as GD, dyslipidemia, chronic hypertension, and obesity, were identified as independent risk factors associated with preeclampsia in patients with SLE. Moreover, patients with SLE in the highest quartile of income had lower odds of developing preeclampsia compared with those in the lowest income quartile, and patients with Medicare or Medicaid had higher odds of preeclampsia compared with patients with private insurance.

### SLE and Risk for CVD

First, our study found that SLE was independently associated with acute adverse CV complications at the time of delivery hospitalization. Previous literature findings suggest that individuals with SLE have worse obstetric outcomes in terms of preeclampsia and preterm birth.^17,18,22^ Studies^13,23,24,25,26^ have found that a chronic inflammatory state was associated with increased risk for preeclampsia and long-term CV events. Our study may represent a significant addition to the existing literature because it found that SLE was associated with acute peripartum CV events, an association that was independent of traditional risk factors and preeclampsia.

We speculate that the association of SLE with acute CV complications may be multifactorial.^9^ Our study provides insights into the cardiometabolic health of individuals with SLE who were older and had a higher prevalence of obesity and obesity-related comorbidities, including GD and PCOS. It may be postulated that the increased prevalence of cardiometabolic risk factors may be associated with the use of steroids in these patients, which has been shown to be associated with a 2-fold higher risk of CV events.^27^ It is important to note that prior studies evaluating the association of immunosuppressive medications with CV complications have found conflicting results, with some suggesting that reducing lupus inflammatory activity may be associated with beneficial outcomes, while others found that these medications were associated with higher rates of CV complications in the long term.^9,23,28^ Our finding that SLE was associated independently with acute CV complications even after adjustment for cardiometabolic risk factors suggests that the underlying inflammatory activity may have a predominant role to play.

### Adverse Temporal Trends of SLE and Associated Cardiometabolic Risk Factors in the US

In this analysis, we report concerning population trends in individuals with SLE in the United States. Our study found an exponential increase in the prevalence of PCOS, GD, and obesity in patients with SLE during a 15-year period from a nationally representative data set. Furthermore, parallel with an increase in the overall prevalence of SLE during delivery admissions, there was an associated increase in cumulative CV events. Our study supports the findings of prior studies^29,30^ that have reported a more than 200% increase in CVD risk factors among individuals of reproductive age. The increased prevalence in temporal trend may also be explained by increased conception rates among individuals with SLE due to better management of the disease in the last decade. To our knowledge, our study provides the most recent data on population-level trends of SLE at pregnancy delivery that have not been described before and may warrant an urgent public health intervention.

### Racial and Socioeconomic Disparities

In addition to reporting on traditional risk factors associated with preeclampsia, our study also found a significant racial and socioeconomic disparity in patients with SLE. In this population, underrepresented racial and ethnicity groups, including Black, Hispanic, and Asian or Pacific Islander individuals, had a higher risk of preeclampsia, similar to racial and ethnic patterns in the non-SLE population.^31^ Similarly, individuals in higher quartiles of income had lower odds of developing preeclampsia, whereas patients with Medicare or Medicaid had higher odds of preeclampsia complications. Socioeconomic status is known to be associated with disparities in preeclampsia-associated CV complications, with pregnant individuals of lower income having an increased risk for maternal mortality.^32^ These findings most likely indicate underlying biases, structural racism, and lack of access of care for socially disadvantaged groups who are undertreated and underscreened.^33,34^ Racial disparities in SLE for premature mortality have already been described,^35^ and our study has now found racial disparities for adverse maternal CV outcomes at delivery for individuals with SLE. Hence, our findings may support urgent measures taken to meet the unmet needs of populations in the US at increased risk of adverse outcomes to reverse trends of increasing maternal mortality.

### Implication for Prevention of CVD in Individuals With GD

Our study findings suggest that individuals with SLE at the time of delivery hospitalization should be counseled on the possible risk of developing acute CV complications. In addition to hypertension, diabetes, obesity, and dyslipidemia, the underlying inflammatory state due to SLE may also need to be managed. Hence, urgent steps may also be needed for prepregnancy screening to identify risk factors and address prevention and treatment of obesity and obesity-related conditions, including PCOS and GD. A multidisciplinary team approach, with guidance from a rheumatologist to address the underlying inflammatory state, is a current practice. Continued close multidisciplinary collaboration at the time of delivery admission may be associated with lower rates of CV complications.^36^

### Resource Use

We found an increase in the length of stay and the associated cost of hospitalization at the time of delivery in patients with a diagnosis of SLE. We postulate that increased hospital resource use may also be a surrogate for increased rates of adverse CV events during hospitalization. Moreover, SLE complications are a source of significant economic burden on the health care system. For example, a 2017 study^37^ estimated that the mean annual direct and indirect cost for SLE in US was $3.9 million to $6.4 million. Our cost analysis suggests a high cumulative cost of SLE hospitalization during delivery and may be helpful information for policy makers.

### Strengths

Our study has several strengths. We analyzed a large, multiethnic, nationally representative sample of the US delivery population. This allowed us to have sufficient statistical power to examine the association of deliveries among individuals with SLE with CV complications over a 15-year period.

### Limitations

Our study has several important limitations that should be considered. The NIS is an administrative claim–based database that uses *ICD-9* and *ICD-10* codes for diagnosis; although we used diagnosis codes less prone to error, coding errors cannot be excluded. There may be undercoding and underreporting of SLE, which may preclude accurate capture of the true disease prevalence in this population. Ascertainment bias is a possibility given that patients with SLE may have been escalated to a higher level of care, which may be associated more diagnostic testing. We were not able to include important variables, such as gestational age at delivery, previous history of preeclampsia or eclampsia, severity of SLE, or prepregnancy body mass index, in our regression model due to the lack of specific *ICD-9* and *ICD-10* codes for these diagnoses. Additionally, we were not able to capture use of medications, such as prepregnancy disease-modifying antirheumatic drugs. There was a change in the methodology of NIS to improve national estimates in 2012 and a change in coding practices from *ICD-9* to *ICD-10* in quarter 4 of 2015 that may have been associated with different estimates of disease prevalence in 2012 or 2015. However, trends we observed were present across the full study period.^38^ Trends in the prevalence of SLE, obesity, GD, and obesity over time may be associated with better capture of these diagnoses over time by *ICD-9* and *ICD-10* coding. Nevertheless, the true prevalence of cardiometabolic risk factors may be underestimated given the reliance on *ICD-9* and *ICD-10* coding for diagnosis. Another limitation is that NIS collects data on inpatient discharges, and each admission is registered as an independent event. NIS samples are not designed to follow up patients longitudinally, so long-term outcomes could not be assessed from the data set used in this study. Only information at the time of hospital delivery was available for analysis, and that has important implications for our study; for instance, PPCM is most likely diagnosed 1 to 4 weeks after delivery. Additionally, as with any observational study, association does not mean causation, and conclusions should be drawn cautiously.

## Conclusions

This cross-sectional study found higher rates of CV complication, including preeclampsia, PPCM, stroke, pulmonary edema, VTE, and cardiac arrhythmia, among individuals giving birth who had SLE compared with those without SLE during delivery hospitalizations in the US over a 15-year period. Moreover, there was increasing prevalence of SLE, CV complications associated with SLE, obesity, and related comorbidities, including GD and PCOS, in the US during delivery hospitalizations. Our findings suggest that a multidisciplinary approach to treatment of pregnant patients with SLE in liaison with a rheumatologist, a cardiologist, and an obstetrician may be associated with improved outcomes. Furthermore, focused studies are needed to best strategize for the prevention and management of acute and long-term pregnancy-associated CV complications among individuals with SLE.

## Notes

Supplement. **eFigure 1.** Central Illustration **eFigure 2.** Study Flow Chart **eFigure 3.** Prevalence of Polycystic Ovary Syndrome, Gestational Diabetes, and Obesity **eMethods 1.** Variables Used in Logistic Regression Analysis to Compute Adjusted Odds of In-Hospital Complications **eMethods 2.** Variables Used in Logistic Regression Analysis to Compute Factors Independently Associated With Preeclampsia in Individuals With Systemic Lupus Erythematosus **eTable 1.** Procedure and Diagnosis Codes Used **eTable 2.** Effect Sizes of Hospitalization Characteristics Among Individuals With vs Without Systemic Lupus Erythematosus **eTable 3.** Factors Associated With Cardiovascular Complications With vs Without Systemic Lupus Erythematosus After Adjustment for Preeclampsia and Eclampsia **eTable 4.** Factors Associated With Cardiovascular Complications After Adjustment for Calendar Year **eTable 5.** Factors Associated With Cardiovascular Complications Among Women Aged 35 Years or Younger **eTable 6.** Factors Associated With Cardiovascular Complications Among Women Older Than 35 Years **eTable 7.** Factors Associated With Cardiovascular Complications After Exclusion of Individuals With Preeclampsia or Eclampsia **eTable 8.** Factors Associated With Cardiovascular Complications After Exclusion of Individuals With Polycystic Ovary Syndrome **eTable 9.** Factors Associated With Cardiovascular Complications After Exclusion of Individuals With Gestational Diabetes **eTable 10.** Factors Associated With Cardiovascular Complications After Exclusion of Individuals With Chronic Kidney Disease, Coronary Artery Disease, or Chronic Heart Failure
