# Supplementary material for: Analysis of Cardiovascular Complications During Delivery Admissions Among Patients With Systemic Lupus Erythematosus, 2004-2019
Source: JAMA Netw Open. 2022 Nov 29;5(11):e2243388. doi: 10.1001/jamanetworkopen.2022.43388 (PMC9709646; doi:10.1001/jamanetworkopen.2022.43388)
Supplement: Supplement. — eFigure 1. Central Illustration eFigure 2. Study Flow Chart eFigure 3. Prevalence of Polycystic Ovary Syndrome, Gestational Diabetes, and Obesity eMethods 1. Variables Used in Logistic Regression Analysis to Compute Adjusted Odds of In-Hospital Complications eMethods 2. Variables Used in Logistic Regression Analysis to Compute Factors Independently Associated With Preeclampsia in Individuals With Systemic Lupus Erythematosus eTable 1. Procedure and Diagnosis Codes Used eTable 2. Effect Sizes of Hospitalization Characteristics Among Individuals With vs Without Systemic Lupus Erythematosus eTable 3. Factors Associated With Cardiovascular Complications With vs Without Systemic Lupus Erythematosus After Adjustment for Preeclampsia and Eclampsia eTable 4. Factors Associated With Cardiovascular Complications After Adjustment for Calendar Year eTable 5. Factors Associated With Cardiovascular Complications Among Women Aged 35 Years or Younger eTable 6. Factors Associated With Cardiovascular Complications Among Women Older Than 35 Years eTable 7. Factors Associated With Cardiovascular Complications After Exclusion of Individuals With Preeclampsia or Eclampsia eTable 8. Factors Associated With Cardiovascular Complications After Exclusion of Individuals With Polycystic Ovary Syndrome eTable 9. Factors Associated With Cardiovascular Complications After Exclusion of Individuals With Gestational Diabetes eTable 10. Factors Associated With Cardiovascular Complications After Exclusion of Individuals With Chronic Kidney Disease, Coronary Artery Disease, or Chronic Heart Failure [file jamanetwopen-e2243388-s001.pdf]

Supplemental Online Content

Zahid S, Mohamed MS, Wassif H, Nazir NT, Khan SS, Michos ED. Analysis of cardiovascular complications during delivery admissions among patients with systemic lupus erythematosus, 2004-2019. *JAMA Netw Open.* 2022;5(11):e2243388. doi:10.1001/jamanetworkopen.2022.43388

eFigure 1. Central Illustration
eFigure 2. Study Flow Chart
eFigure 3. Prevalence of Polycystic Ovary Syndrome, Gestational Diabetes, and Obesity
eMethods 1. Variables Used in Logistic Regression Analysis to Compute Adjusted Odds of In-Hospital Complications
eMethods 2. Variables Used in Logistic Regression Analysis to Compute Factors Independently Associated With Preeclampsia in Individuals With Systemic Lupus Erythematosus
eTable 1. Procedure and Diagnosis Codes Used
eTable 2. Effect Sizes of Hospitalization Characteristics Among Individuals With vs Without Systemic Lupus Erythematosus
eTable 3. Factors Associated With Cardiovascular Complications With vs Without Systemic Lupus Erythematosus After Adjustment for Preeclampsia and Eclampsia
eTable 4. Factors Associated With Cardiovascular Complications After Adjustment for Calendar Year
eTable 5. Factors Associated With Cardiovascular Complications Among Women Aged 35 Years or Younger
eTable 6. Factors Associated With Cardiovascular Complications Among Women Older Than 35 Years
eTable 7. Factors Associated With Cardiovascular Complications After Exclusion of Individuals With Preeclampsia or Eclampsia
eTable 8. Factors Associated With Cardiovascular Complications After Exclusion of Individuals With Polycystic Ovary Syndrome
eTable 9. Factors Associated With Cardiovascular Complications After Exclusion of Individuals With Gestational Diabetes
eTable 10. Factors Associated With Cardiovascular Complications After Exclusion of Individuals With Chronic Kidney Disease, Coronary Artery Disease, or Chronic Heart Failure

This supplemental material has been provided by the authors to give readers additional information about their work.

© 2022 Zahid S et al. *JAMA Network Open.*

eFigure 1. Central Illustration

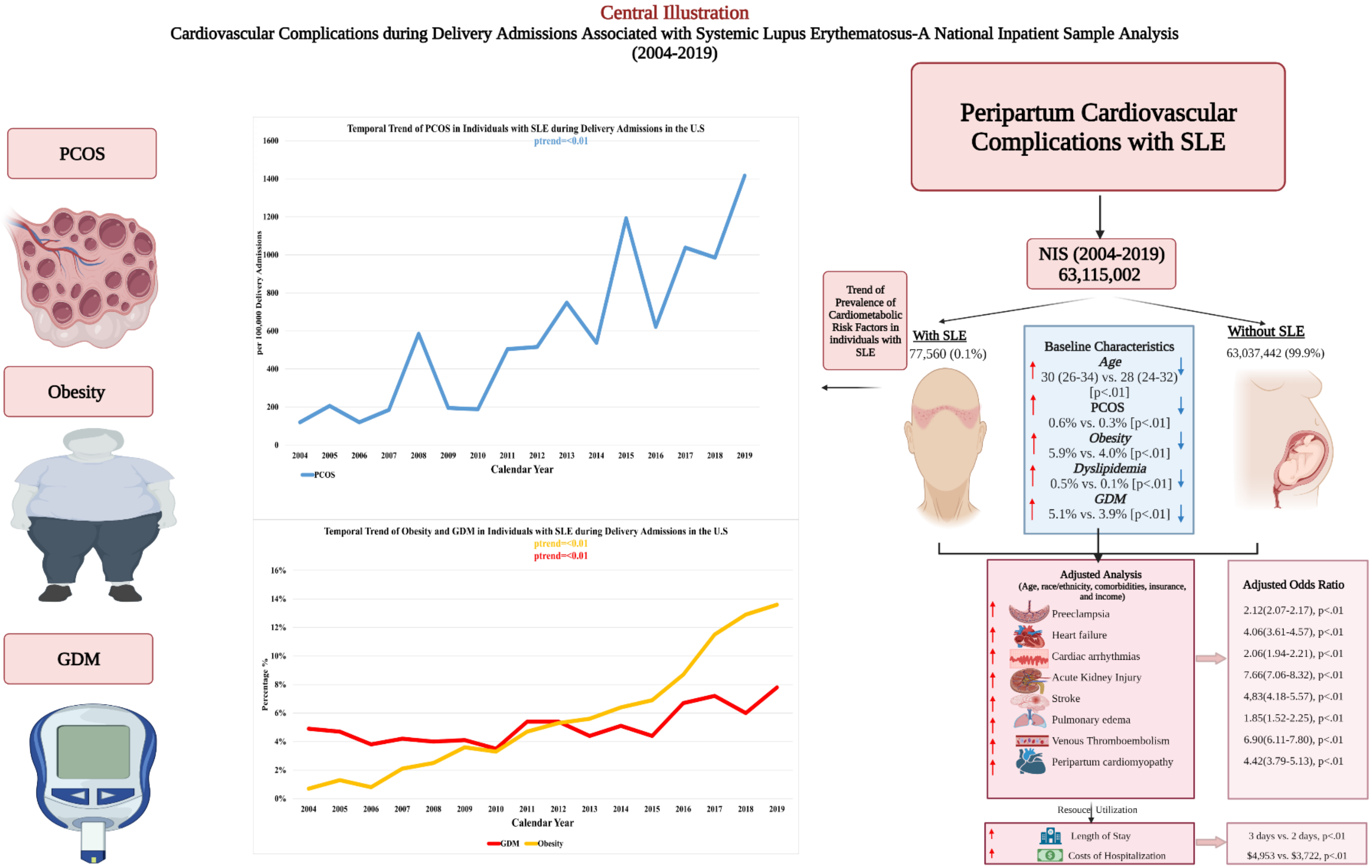

© 2022 Zahid S et al. *JAMA Network Open.*

eFigure 2. Study Flow Chart

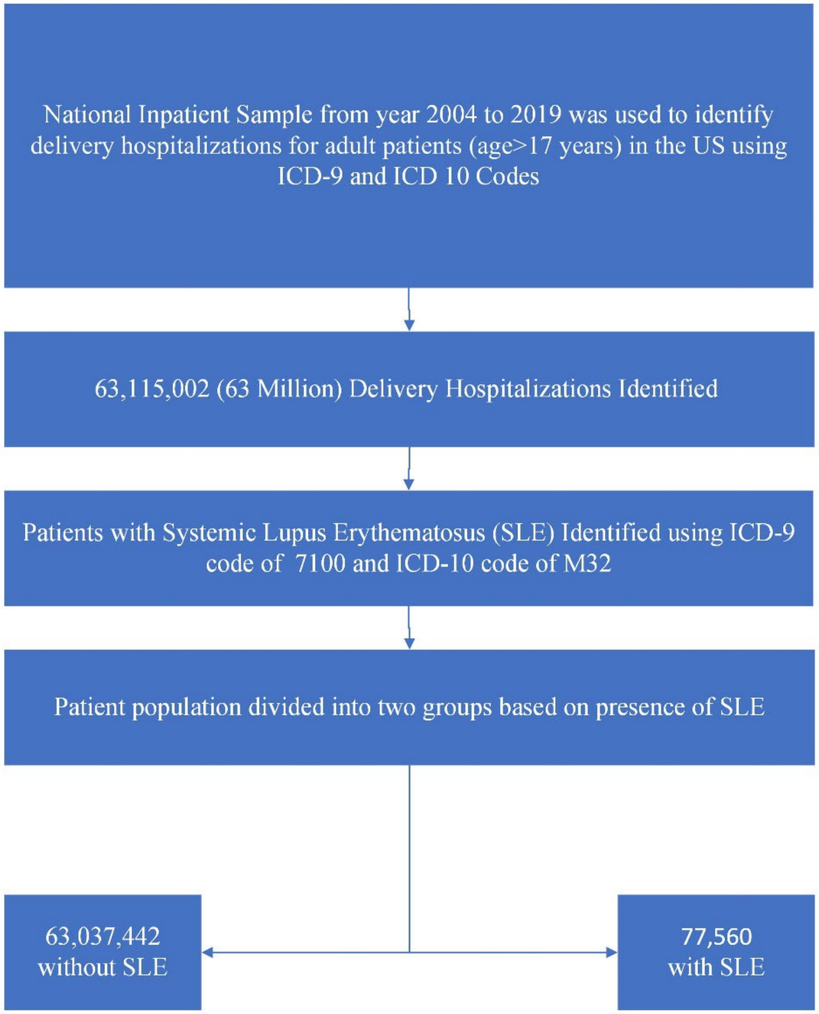

© 2022 Zahid S et al. *JAMA Network Open.*

eFigure 3. Prevalence of Polycystic Ovary Syndrome, Gestational Diabetes, and Obesity

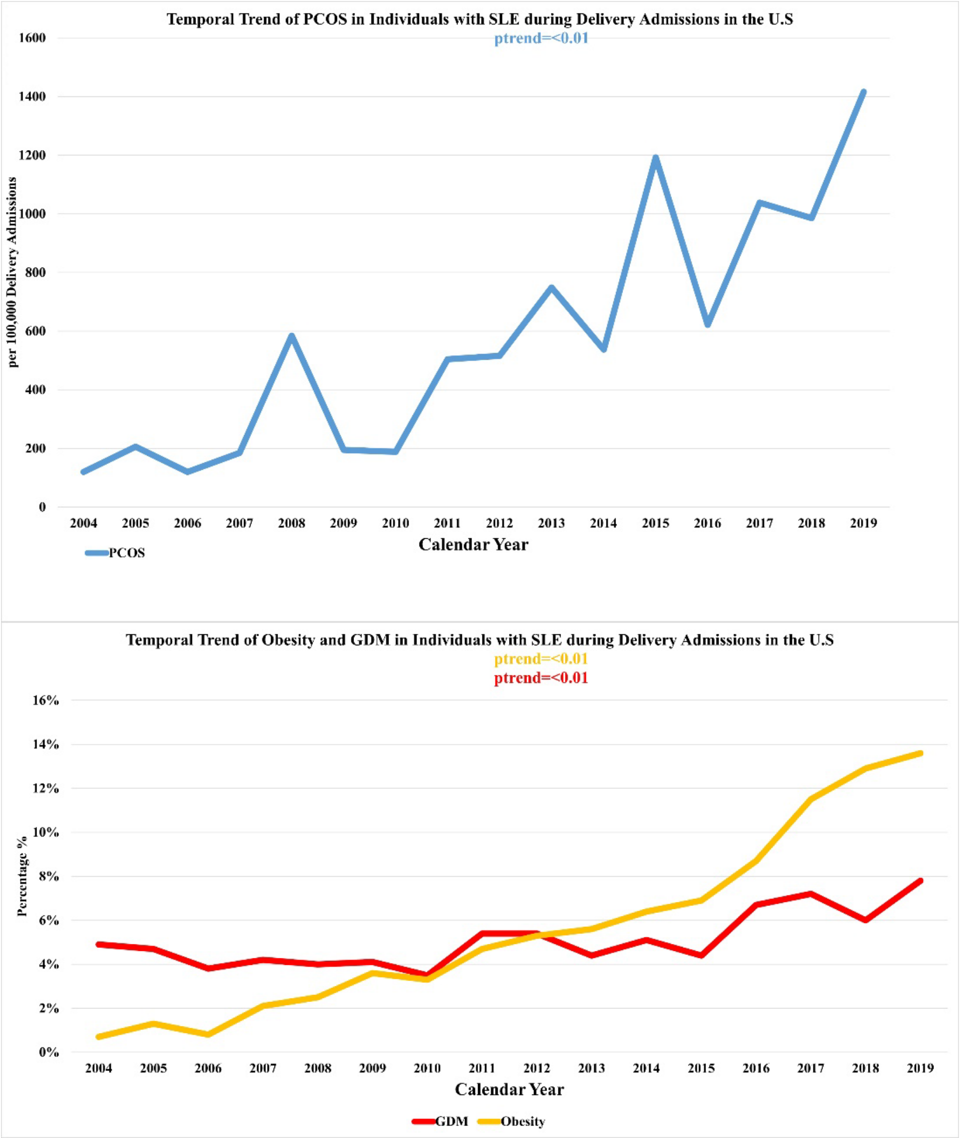

Abbreviations: US: United States, PCOS: Polycystic Ovary Syndrome, GDM: Gestational Diabetes Mellitus

© 2022 Zahid S et al. *JAMA Network Open.*

eMethods 1. Variables Used in Logistic Regression Analysis to Compute Adjusted Odds of In-Hospital Complications

1- Age
2- Race
3- Chronic Hypertension
4- Gestational Diabetes Mellitus
5- Dyslipidemia
6- Congestive Heart Failure
7- Chronic Kidney Disease
8- Coronary Artery Disease
9- Obesity
10- PCOS
11- Smoking
12- Multiple Gestation
13- Cesarean Delivery
14- Median Household Income
15- Primary Insurance

© 2022 Zahid S et al. *JAMA Network Open.*

eMethods 2. Variables Used in Logistic Regression Analysis to Compute Factors Independently Associated With Preeclampsia in Individuals With Systemic Lupus Erythematosus

1- Age
2- Race
3- Chronic Hypertension
4- Gestational diabetes mellitus
5- Dyslipidemias
6- Polycystic Ovary Syndrome
7- Obesity
8- Primary Insurance
9- Median Household Income

© 2022 Zahid S et al. *JAMA Network Open.*

eTable 1. Procedure and Diagnosis Codes Used

Variables	ICD-10	ICD-9
**Delivery**	10D0, 10E0, O60, O61, O62, O63, O64, O65, O66, O67, O68, O69, O70, O71, O72, O73, O74, O75, O76, O77, O80, O82, Z37, Z38	72, 73,75, 650, 651, 652, 653, 654, 655, 656, 657, 658, 659, 660, 662, 663, 664, 665, 666, 667, 668, 669, V27
**Systemic Lupus Erythematosus**	M32	7100
**Gestational Diabetes Mellitus**	648.8	0244x
**Polycystic Ovary Syndrome**	E282	256.4
**Preeclampsia/ Eclampsia**	O1400, O140, O1402, O1403, O1404, O1405, O1490, O1492, O1493, O1494, O1495, O149, O141, O1410, O1412, O1413, O1414, O1415, O11, O111, O112, O113, O114, O115, O119, O142, O1420, O1422, O1423, O1424,O1425, O15, O150, O1500, O1502, O1503, O151, O152, O159	64241, 64242, 64243, 64244, 64250, 64251, 64252, 64253, 64254, 64270, 64271, 64272, 64273, 64274, 64260, 64261, 64262, 64263, 64264
**Peripartum Cardiomyopathy**	O903	6745
**Heart Failure**	I5021, I5031, I5033, I5041, I5043	42821, 42823, 42831, 42841, 42843
**Acute Kidney Injury**	N17	584,
**Stroke**	I60, I61, I62, I63, I650, I688, O873, O2250, O2251, O2252	430, 431, 432, 433, 437, 6715

© 2022 Zahid S et al. *JAMA Network Open.*

**Pulmonary edema**	J810, J811, I501	514
**Cardiac Arrhythmias**	Z450, Z950, T821, R001, R008, R000,<br>I459, I456, I441, I442, I443, I47, I48,<br>I49	4260, 42613, 4267, 4269, 42610, 42612, 4270, 4271, 4272, 4273, 4274, 4276, 4277, 4278, 4279, 7850, 99601, 99604, V450, V533
**Acute Venous Thromboembolism**	I82210, I82220, I82290, I824, I8241, I8242, I8243. I8244, I8245, I8246, I8249, I824Y, I824Z, I826, I8261, I8262, I82A1, I82B1, I82C1, I8281, I82890, I8290	4534, 45340, 45341, 45342, 4538, 45381, 45382, 45383, 45384, 45385, 45386, 45387, 45389

ICD: International Classification of Diseases

© 2022 Zahid S et al. *JAMA Network Open.*

eTable 2. Effect Sizes of Hospitalization Characteristics Among Individuals With vs Without Systemic Lupus Erythematosus

Variables (continuous)*	Beta coefficient (95% CI)
Resource Utilization	
Length of stay (days)	1.23 (1.21-1.24)
Cost of hospitalization ($)	2,470 (2,436-2,504)
Patient Demographics	
Age (years)	1.62 (1.58-1.66)
**Variables (categorical)****	**OR (95% CI)**
Patient Demographics	
Race/Ethnicity	
White	0.88 (0.86-0.89)
Black	1.92 (1.89-1.95)
Hispanic	0.86 (0.84-0.87)
Asian or Pacific Islander	0.98 (0.95-1.01)
Native American	1.08 (0.99-1.17)
Other	0.78 (0.77-0.81)
Hospital Regions	
Northeast	1.17 (1.15-1.19)
Midwest	0.86 (0.85-0.88)
South	1.04 (1.03-1.06)
West	0.95 (0.93-0.96)
Pre-existing co-morbidities	
PCOS	1.98 (1.81-2.17)
GDM	1.35 (1.31-1.39)
Dyslipidemia	3.31 (3.00-3.65)
Chronic Hypertension	7.93 (7.67-8.19)
Heart Failure	12.62 (11.64-13.69)
Chronic Kidney Disease	81.61 (76.09-87.53)

© 2022 Zahid S et al. *JAMA Network Open.*

**Coronary Artery Disease**	20.68 (17.86-23.94)
**Obesity**	1.51 (1.46-1.55)
**Smoking**	1.21 (1.15-1.27)
Obstetric Characteristics	
**Multiple Gestation**	1.40 (1.34-1.47)
**Cesarean delivery**	1.55 (1.52-1.57)
**Preterm Birth**	2.24 (2.20-2.84)
**Still Birth**	2.48 (2.34-2.62)
Socioeconomic Characteristics	
Median Household Income	
**0-25th percentile**	0.99 (0.97-1.01)
**26-50th percentile**	0.91 (0.88-0.91)
**51-75th percentile**	0.99 (0.97-1.01)
**76-100th percentile**	1.14 (1.12-1.16)
Primary Insurance	
**Medicare**	7.63 (7.38-7.88)
**Medicaid**	0.80 (0.79-0.81)
**Private insurance**	1.09 (1.08-1.11)
**Self-Pay**	0.49 (0.46-0.52)
**No Charge**	0.53 (0.41-0.67)
**Other**	1.15 (1.11-1.20)

* Effect sizes for continuous variables of length of stay and cost of hospitalization for SLE in comparison to non-SLE group are reported as coefficients and their 95% confidence intervals. For example, for length of stay, this means that a patient with SLE will have 1.23 more admitted days than a patient without SLE. For cost, it means a patient with SLE will have $2470 more cost compared to admitted patient without SLE. 95% confidence intervals are in the same units. **Effect sizes for categorical variables are reported as odds ratios and their 95% confidence interval. The comparison group for each variable is non-SLE patients. For example SLE patients in this cohort were 22% less likely to be White compared to non-SLE patients (OR 0.88). Another example, SLE patients were 7.9-fold more likely to have chronic hypertension compared to non-SLE patients.

© 2022 Zahid S et al. *JAMA Network Open.*

eTable 3. Factors Associated With Cardiovascular Complications With vs Without Systemic Lupus Erythematosus After Adjustment for Preeclampsia and Eclampsia

**Variables**	**aOR**	**p-value**
**Peripartum Cardiomyopathy**	1.71 (1.45-2.02)	<0.01
**Heart Failure**	1.20 (0.99-1.45)	0.07
**Acute Kidney Injury**	6.76 (6.24-7.33)	<0.01
**Stroke**	4.06 (3.51-4.69)	<0.01
**Pulmonary Edema**	1.31 (1.17-1.58)	<0.01
**Cardiac Arrhythmias**	1.89 (1.78-2.02)	<0.01
**Venous Thromboembolism**	6.35 (5.62-7.19)	<0.01

aOR: Adjusted Odds Ratio; SLE: Systemic Lupus Erythematosus
Adjusted for age, race/ethnicity, chronic hypertension, dyslipidemia, heart failure, chronic kidney disease, coronary artery disease, GDM, obesity, PCOS, smoking, multiple gestation, Cesarean delivery, median household income, primary insurance with additional adjustment of preeclampsia and eclampsia

© 2022 Zahid S et al. *JAMA Network Open.*

eTable 4. Factors Associated With Cardiovascular Complications After Adjustment for Calendar Year

Variables	aOR	p-value
**Preeclampsia**	1.99 (1.95-2.04)	<0.01
**Peripartum Cardiomyopathy**	1.64 (1.39-1.93)	<0.01
**Heart Failure**	1.18 (0.97-1.43)	0.11
**Acute Kidney Injury**	7.27 (6.69-7.89)	<0.01
**Stroke**	4.69 (4.06-5.42)	<0.01
**Pulmonary Edema**	1.54 (1.26-1.88)	<0.01
**Cardiac Arrhythmias**	1.87 (1.75-1.99)	<0.01
**Venous Thromboembolism**	6.38 (5.55-7.10)	<0.01

Adjusted for age, race/ethnicity, chronic hypertension, dyslipidemia, heart failure, chronic kidney disease, coronary artery disease, GDM, obesity, PCOS, smoking, multiple gestation, Cesarean delivery, median household income, primary insurance with additional adjustment for calendar year.

© 2022 Zahid S et al. *JAMA Network Open.*

eTable 5. Factors Associated With Cardiovascular Complications Among Women Aged 35 Years or Younger

Variables	aOR	p-value
**Preeclampsia**	2.15 (2.09-2.21)	<0.01
**Peripartum Cardiomyopathy**	1.61 (1.33-1.95)	<0.01
**Heart Failure**	1.12 (0.89-1.41)	0.33
**Acute Kidney Injury**	7.87 (7.16-8.64)	<0.01
**Stroke**	3.95 (2.28-4.74)	<0.01
**Pulmonary Edema**	1.36 (1.06-175)	0.02
**Cardiac Arrhythmias**	1.91 (1.78-2.06)	<0.01
**Venous Thromboembolism**	7.89 (6.85-9.08)	<0.01

aOR: Adjusted Odds Ratio; SLE: Systemic Lupus Erythematosus
Adjusted for age, race/ethnicity, chronic hypertension, dyslipidemia, heart failure, chronic kidney disease, coronary artery disease, GDM, obesity, PCOS, smoking, multiple gestation, Cesarean delivery, median household income and primary insurance

© 2022 Zahid S et al. *JAMA Network Open.*

eTable 6. Factors Associated With Cardiovascular Complications Among Women Older Than 35 Years

Variables	aOR	p-value
**Preeclampsia**	1.80 (1.71-1.91)	<0.01
**Peripartum Cardiomyopathy**	1.73 (1.27-2.36)	<0.01
**Heart Failure**	1.58 (1.02-2.46)	0.04
**Acute Kidney Injury**	4.97 (4.12-5.99)	<0.01
**Stroke**	6.41 (5.08-8.09)	<0.01
**Pulmonary Edema**	1.96 (1.41-2.73)	<0.01
**Cardiac Arrhythmias**	2.03 (1.77-2.33)	<0.01
**Venous Thromboembolism**	4.18 (3.22-5.42)	<0.01

Adjusted for age, race/ethnicity, chronic hypertension, dyslipidemia, heart failure, chronic kidney disease, coronary artery disease, GDM, obesity, PCOS, smoking, multiple gestation, Cesarean delivery, median household income and primary insurance.

© 2022 Zahid S et al. *JAMA Network Open.*

eTable 7. Factors Associated With Cardiovascular Complications After Exclusion of Individuals With Preeclampsia or Eclampsia

Variables	aOR	p-value
**Peripartum Cardiomyopathy**	1.33 (1.08-1.63)	0.01
**Heart Failure**	1.29 (1.03-1.62)	0.02
**Acute Kidney Injury**	7.98 (7.12-8.95)	<0.01
**Stroke**	4.11 (3.39-4.99)	<0.01
**Pulmonary Edema**	2.22 (1.69-2.91)	<0.01
**Cardiac Arrhythmias**	1.95 (1.81-2.09)	<0.01
**Venous Thromboembolism**	6.03 (5.23-6.95)	<0.01

Adjusted for age, race/ethnicity, chronic hypertension, dyslipidemia, heart failure, chronic kidney disease, coronary artery disease, GDM, obesity, PCOS, smoking, multiple gestation, Cesarean delivery, median household income and primary insurance.

© 2022 Zahid S et al. *JAMA Network Open.*

eTable 8. Factors Associated With Cardiovascular Complications After Exclusion of Individuals With Polycystic Ovary Syndrome

Variables	aOR	p-value
**Preeclampsia**	2.08 (2.03-2.13)	<0.01
**Peripartum Cardiomyopathy**	2.08 (2.03-2.13)	<0.01
**Heart Failure**	1.17 (0.96-1.43)	0.11
**Acute Kidney Injury**	7.47 (6.88-8.11)	<0.01
**Stroke**	4.67 (4.04-5.40)	<0.01
**Pulmonary Edema**	1.56 (1.28-1.91)	<0.01
**Cardiac Arrhythmias**	1.97 (1.85-2.10)	<0.01
**Venous Thromboembolism**	6.58 (5.81-7.45)	<0.01

Adjusted for age, race/ethnicity, chronic hypertension, dyslipidemia, heart failure, chronic kidney disease, coronary artery disease, GDM, obesity, smoking, multiple gestation, Cesarean delivery, median household income and primary insurance.

© 2022 Zahid S et al. *JAMA Network Open.*

eTable 9. Factors Associated With Cardiovascular Complications After Exclusion of Individuals With Gestational Diabetes

Variables	aOR	p-value
**Preeclampsia**	2.15 (2.10-2.21)	<0.01
**Peripartum Cardiomyopathy**	1.52 (1.29-1.81)	<0.01
**Heart Failure**	1.15 (0.94-1.40)	0.17
**Acute Kidney Injury**	7.38 (6.77-8.04)	<0.01
**Stroke**	4.46 (3.84-5.19)	<0.01
**Pulmonary Edema**	1.47 (1.19-1.81)	<0.01
**Cardiac Arrhythmias**	1.97 (1.84-2.11)	<0.01
**Venous Thromboembolism**	6.83 (6.03-7.75)	<0.01

Adjusted for age, race/ethnicity, chronic hypertension, dyslipidemia, heart failure, chronic kidney disease, coronary artery disease, obesity, PCOS, smoking, multiple gestation, Cesarean delivery, median household income and primary insurance.

© 2022 Zahid S et al. *JAMA Network Open.*

eTable 10. Factors Associated With Cardiovascular Complications After Exclusion of Individuals With Chronic Kidney Disease, Coronary Artery Disease, or Chronic Heart Failure

Variables	aOR	p-value
**Preeclampsia**	2.08 (2.03-2.13)	<0.01
**Peripartum Cardiomyopathy**	1.82 (1.54-2.14)	<0.01
**Heart Failure**	1.17 (0.96-1.43)	0.12
**Acute Kidney Injury**	7.60 (7.00-8.25)	<0.01
**Stroke**	4.75 (4.12-5.49)	<0.01
**Pulmonary Edema**	1.66 (1.37-2.02)	<0.01
**Cardiac Arrhythmias**	1.98 (1.85-2.11)	<0.01
**Venous Thromboembolism**	6.72 (5.95-7.81)	<0.01

Adjusted for age, race/ethnicity, chronic hypertension, dyslipidemia, GDM, obesity, PCOS, smoking, multiple gestation, Cesarean delivery, median household income and primary insurance.

© 2022 Zahid S et al. *JAMA Network Open.*
